# RECRUITING AND ENGAGING TRANSGENDER, NON-BINARY, AND INTERSEX OLDER ADULTS IN AGING RESEARCH

**DOI:** 10.1093/geroni/igac059.460

**Published:** 2022-12-20

**Authors:** Nik Lampe

**Affiliations:** Vanderbilt University, Nashville, Tennessee, United States

## Abstract

Aging research often erases sex and gender variation, thus ignoring the experiences of older transgender, non-binary, and intersex (TNBI) populations. At the same time, researchers have faced unique methodological challenges with recruiting and engaging with TNBI older patient populations in sexual and gender minority (SGM) aging research. Using data from 51 semi-structured individual interviews with TNBI older Americans, I explore methodological approaches for effectively recruiting and engaging TNBI older respondents. I describe the importance of disclosing researcher positionality and understanding community discourse (e.g., intersex status as a sex identity vs. a medical condition) when recruiting and engaging with TNBI older adults. Finally, I share why some traditional recruitment approaches in SGM aging research (e.g., purposive/snowball sampling techniques) may not be as effective towards TNBI older adults while offering some alternative techniques for meaningful recruitment and engagement opportunities.

